# Monkeypox infection presenting as genital rash, Australia, May 2022

**DOI:** 10.2807/1560-7917.ES.2022.27.22.2200411

**Published:** 2022-06-02

**Authors:** Yael Hammerschlag, Gina MacLeod, Georgina Papadakis, Asiel Adan Sanchez, Julian Druce, George Taiaroa, Ivana Savic, Jamie Mumford, Jason Roberts, Leon Caly, Deborah Friedman, Deborah A Williamson, Allen C Cheng, James H McMahon

**Affiliations:** 1 Department of Infectious Diseases, Alfred Hospital and Monash University, Melbourne, Australia; 2 Victorian Infectious Diseases Reference Laboratory, Royal Melbourne Hospital at the Peter Doherty Institute for Infection and Immunity, Melbourne, Australia; 3 Northside Clinic, North Fitzroy, Australia; 4 Department of Infectious Diseases, University of Melbourne, Melbourne, Australia; 5 Public Health Division, Victorian Government Department of Health, Melbourne, Australia; 6 Department of Infectious Diseases, Monash Medical Centre, Melbourne, Australia

**Keywords:** monkeypox, viral infections, HIV infection, imported viral diseases

## Abstract

Rapid diagnosis and whole genome sequencing confirmed a case of monkeypox in an HIV-positive individual receiving antiretroviral therapy. The patient had a normal CD4+ T-cell count and suppressed HIV viral load and presented with a genital rash in Melbourne, Australia after return from Europe in May 2022. He subsequently developed systemic illness and disseminated rash and 11 days after symptom onset, he was hospitalised to manage painful bacterial cellulitis of the genital area.

A rapidly emerging outbreak of monkeypox infection in over 20 countries from Europe, North and South America and the Middle East commenced in May 2022 [1]. The majority of cases had no travel history to endemic areas in central and west Africa, were diagnosed through primary care and sexual health services and were mainly reported in men who have sex with men (MSM). Here we present a case of monkeypox infection in an individual diagnosed in Australia after return from Europe.

## Case description

An HIV-positive man in his 30s receiving co-formulated Abacavir, Lamivudine and Dolutegravir and with a CD4 + T-cell count above 700 cells/mm^3^ (normal range 410–1,545 cells/mm^3^) and HIV viral load < 100 copies/mL, visited a primary care doctor after his return from Europe to Melbourne, Australia. He reported onset of a genital rash 8 days earlier. The rash had started 5 days after he reported unprotected insertive anal intercourse with four casual male partners in Europe. The initial symptoms were painless white pustules on the penis that became painful and pruritic. He reported that he developed a fever and malaise 3 days after the first appearance of the penile rash and over the subsequent 5 days, the rash disseminated to his trunk, then more sparingly to the face and limbs while the genital lesions crusted over.

**Figure 1:**
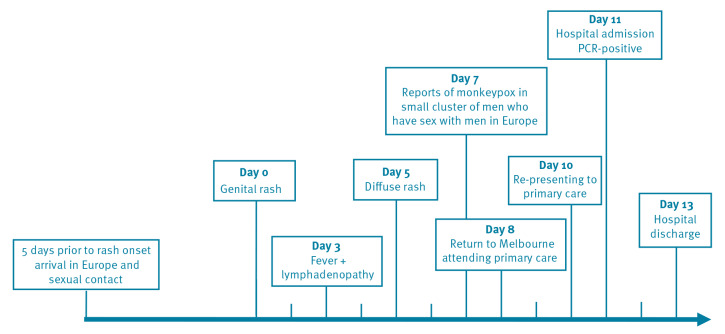
Timeline of exposure and symptoms of monkeypox case imported to Australia from Europe, May 2022

On the day of his initial visit to his primary care doctor, 8 days after symptom onset, he was treated presumptively for gonorrhea and chlamydia with intramuscular ceftriaxone and oral doxycycline. On day 10 after symptom onset, he was assessed again by his primary care doctor for increased genital pain and commenced oral cephalexin for superimposed bacterial cellulitis of his genitals. On day 11 after the onset of rash, his primary care doctor who was aware of a cluster of monkeypox cases reported in MSM in Europe from media reports, referred the patient for inpatient hospital care due to concern for monkeypox and for pain management (Figure 1).

**Figure 2:**
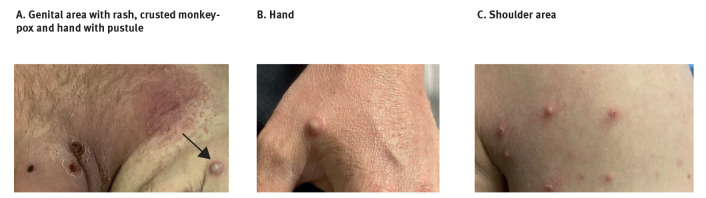
Monkeypox lesions at hospitalisation on day 11 post symptom onset of monkeypox case imported to Australia from Europe, May 2022

On admission to hospital, the patient was managed with contact and airborne precautions in a single room with negative pressure ventilation. These precautions were undertaken as the diagnosis was unclear and varicella zoster virus infection was a potential differential diagnosis requiring airborne precautions. When examined in hospital, the penile lesions had largely formed scabs and the lesions on hands and lower limbs were painless papular pustules. Lesions on the upper limbs and trunk were in various vesicular and crusted states (Figure 2). He was diagnosed with superimposed bacterial cellulitis of the penile shaft and lower central abdomen and treated with intravenous cephazolin and oral analgesia. His cellulitis and pain improved, and he was discharged on day 13 post symptom onset. On discharge from hospital his lesions were still in multiple stages of evolution, some having formed scabs and some still in the early vesicular and pustular phase.

The black arrow indicates hand with pustule.

## Laboratory findings

Routine blood tests (full blood exam, biochemistry liver function tests) performed when admitted to hospital were all within normal limits and a C-reactive protein was mildly elevated at 12 mg/L (reference range: 0–5 mg/L). Test results for varicella zoster virus, herpes simplex virus, *Chlamydia trachomatis* and *Neisseria gonorrhoea* by standard methods using real-time PCR (RT-PCR) from urine and rectal and throat swabs, were all negative. Syphilis serology showed a rapid plasma reagin (RPR) titre of 8 and 4 when checked on two separate occasions, which represented over a 4-fold drop in RPR from when he was treated for syphilis 8 months prior, in 2021.

**Figure 3:**
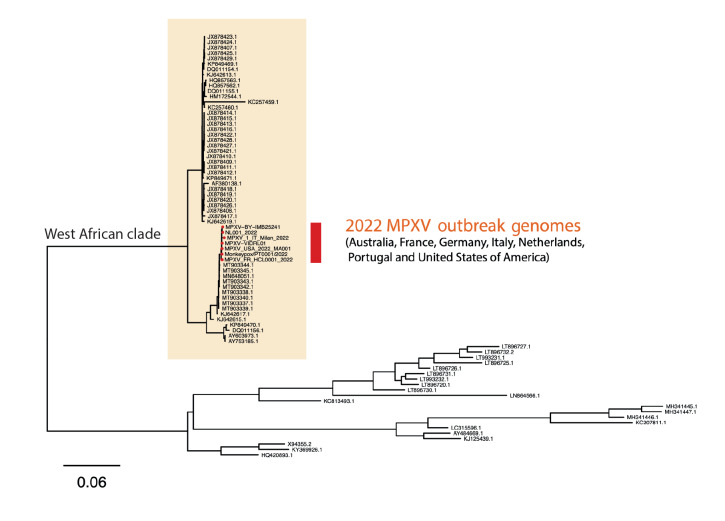
Whole genome sequencing and phylogenetic analysis of monkeypox viruses of the West African clade shows distinct clustering of MPXV-VIDRL01 imported from Europe to Australia with other global outbreak sequences, May 2022

Swabs taken from deroofed skin lesions on the hand, calf and trunk in addition to combined nose throat swabs on the day of hospital admission, were all positive for monkeypox virus using previously described conventional [2] and in-house RT-PCR assays for orthopox and monkeypox viruses (described in the Supplementary material). Whole genome sequencing performed as described in the Supplementary material of DNA derived from the skin lesions resulted in the complete recovery of the entire monkeypox genome (MPXV-VIDRL01, Genbank_ID ON631963) with phylogenetic analysis revealing clustering with other monkeypox virus sequences from the May 2022 outbreak in Europe and the United States (Figure 3).

Phylogenetic relatedness was assessed between available MPXV genomes, with those in NCBI databases supplemented with additional draft genomes deposited on virological.org; using MAFFT (7.475) for alignment followed by IQTREE (2.2.0-beta) inference with a GTR substitution model and tree visualisation using ggtree (2.0.4).

**Figure 4:**
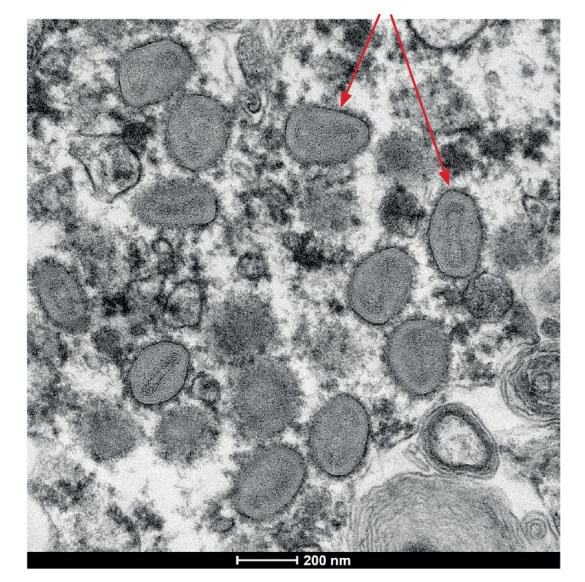
Electron micrograph of pox-like virus particles from thin sections in infected Vero/hSLAM cells of monkeypox case imported to Australia from Europe, May 2022

In addition, monkeypox virus was isolated from swabs of lesions on the hand and abdomen in Vero/hSLAM cells, and the virus was visualised through thin section electron microscopy (Figure 4).

The red arrows are pointing at two of the monkeypox virions.

A description of how the electron microscopy was performed can be found in the Supplementary material.

## Discussion

Monkeypox is a zoonotic disease endemic to Central and West Africa and not historically associated with sexual contact [3,4]. Classical descriptions of monkeypox depict patients initially presenting with a fever and systemic symptoms followed by a rash that is most commonly monomorphic with a centrifugal distribution (concentrated on face and distal extremities) [3,5]. The initial presentation of a penile rash in our patient suggests close physical contact during sexual contact as the route of acquisition. Genital rash is also being reported in the current outbreak of monkeypox in several countries outside the endemic areas in Africa, mainly in MSM [1,6,7]. The clinical presentation in our patient was atypical, firstly due to the presence of rash exclusively on the site of sexual contact 3 days before developing fever and secondly, after the rash disseminated, due to the preponderance for lesions in a central distribution with the least lesions present on the face and extremities. Unfortunately, contact tracing of casual sexual partners, who were the likely source of monkeypox, was not possible due to a lack of contact information.

Potential sexual transmission is suggested by the initial presentation of penile lesions at the site where sexual contact occurred before the systemic illness. However, establishing if monkeypox can be sexually transmitted will require larger studies combining clinical, epidemiological, and detailed virological analyses including phylogeny of transmitted viral strains. Whether or not the HIV infection was contributory to the clinical course in the patient is unknown, but it may be possible that advanced or uncontrolled HIV infection could lead to more severe outcomes. Hospitalisation of our patient was not for severe manifestations of the monkeypox viral infection but to enable diagnostic evaluation, pain management and treatment of bacterial superinfection. His normal CD4+ T-cell count and supressed HIV viral load on antiretroviral therapy were potentially important factors in preventing more severe outcomes from his monkeypox infection.

Monkeypox should be considered in the differential of a vesicular or pustular genital rash and requires prompt diagnosis and a public health response. This includes tracing close contacts and consideration of interventions such as isolation and post-exposure prophylaxis with smallpox vaccine if indicated.

## Supplementary Data

204000 Supplementary Material
