# Supplementary material for: Monkeypox infection presenting as genital rash, Australia, May 2022
Source: Euro Surveill. 2022 Jun 2;27(22):2200411. doi: 10.2807/1560-7917.ES.2022.27.22.2200411 (PMC9164678; doi:10.2807/1560-7917.ES.2022.27.22.2200411)
Supplement: Supplementary Material [file 22-00411_MCMAHON_Supplementary_material.pdf]

Supplementary material

This supplementary material is hosted by *Eurosurveillance* as supporting information alongside the article 'Monkeypox infection presenting as genital rash, Australia, May 2022', on behalf of the authors, who remain responsible for the accuracy and appropriateness of the content. The same standards for ethics, copyright, attributions and permissions as for the article apply. Supplements are not edited by *Eurosurveillance* and the journal is not responsible for the maintenance of any links or email addresses provided therein.

Methods

Real-time PCR detection of monkeypox virus

A TaqMan RT-PCR assay (20ul final volume) comprised of 4 µl gDNA, 10 µl Primer Design PrecisonPLUS qPCR Master Mix (PrimerDesign, Camberley, UK) 1 µM Forward (5'–TCT AAA AAA ATA GGT GAT GAT GCA ACT-3'), 1 µM Reverse (5'- AGA ATA ATG GAA TTG GGC TCC TT –3') primers and 0.2 µM pan-orthopox (5'–VIC- ATA CCA AGC ACT CAT AAC AA-MGBNFQ–3') and Monkeypox specific (5'-FAM-AGT CGA AAT AAT ACA AAT TAC TA-MGBNFQ-3') probes targeting the Orthopox Hemagglutinin (B2R) gene as 2 separate reactions. The real-time RT-PCR assay was performed on an Applied Biosystems ABI 7500 Fast Real-time PCR machine (Applied Biosystems, Foster City, CA, USA) with cycling conditions of 95°C for 2 min, followed by 45 cycles of 95°C for 5 s, 60°C for 30 s.

Metagenomic whole genome sequencing

Total nucleic acid extraction was performed from MPXV positive clinical skin swab samples using the QIAamp Viral RNA mini kit (Qiagen, Hilden, Germany) and eluted into 60ul. DNA concentration was determined using the Qubit dsDNA HS assay kit (Thermofisher, Waltham, MA, USA) before whole genome sequencing library preparation using the Illumina DNA Prep with Tagmentation Kit (Illumina, San Diego, CA, USA). Sequencing was performing using a Mid Output v2.5 2x150bp cartridge on a NextSeq 550 instrument (Illumina, San Diego, CA, USA). Reads were subsequently aligned to the reference MPXV-UK_P3 (MT903345) using minimap2 (2.24) to derive a consensus genome. This genome was further curated based on *de novo* assembly of the minimap2 MPXV-aligned reads using spades (3.14.1) and careful inspection of relevant read alignments. The completed genome (197,443 nucleotides) (Genbank_ID ON631963) had a resultant mean coverage of >240 fold. Phylogenetic relatedness was assessed between available MPXV genomes, with those in NCBI databases

supplemented with additional draft genomes deposited on virological.org; using MAFFT (7.475) for alignment followed by IQTREE (2.2.0-beta) inference with a GTR substitution model and tree visualisation using ggtree (2.0.4).

Electron Microscopy

Thin-sectioning of cell culture-derived suspensions was performed after inactivation in 2% glutaraldehyde for 34 h at 4ºC. Cell suspensions were clarified by centrifugation at 1,500g for 10 minutes, supernatant removed and pelleted cellular material retained for thin section transmission electron microscopy. Pelleted cellular material was enrobed in 2% agarose and processed using a rapid microwave-based, reduced osmium teroxide, en bloc staining adapted from Laue 2010. Briefly, enrobed cells were fixed and stained en bloc using a 1% reduced osmium tetroxide, 0.1% tannic acid and 2% Uranyl Acetate protocol. Fixed and stained cells were then passed through a gradient series of dehydrating ethanol solutions, then transitioned to 100% propylene oxide for embedding in Spurr's resin. Embedded samples were polymerised at 75ºC for 12 h, then thin-sectioned using a Leica UC7 ultramicrotome. 70nm sections were transferred to copper 100-mesh electron microscopy grids and post stained in a 2% lead citrate solution for 5 min. The sections were examined using an FEI T12 Spirit electron microscope operating at an acceleration voltage of 80 keV. Electron micrographs were collected using an FEI Eagle 16MP CCD camera.
